# Left Atrial Strain in Patients with Chronic Heart Failure with Preserved Ejection Fraction: A Narrative Review

**DOI:** 10.3390/life15020313

**Published:** 2025-02-17

**Authors:** Dana Emilia Man, Alexandru Catalin Motofelea, Valentina Buda, Dana Emilia Velimirovici, Olivia Bodea, Daniel Marius Duda-Seiman, Constantin Tudor Luca, Simona-Ruxanda Dragan

**Affiliations:** 1 University Clinic of Internal Medicine and Ambulatory Care, Prevention and Cardiovascular Recovery, Department VI—Cardiology, “Victor Babes” University of Medicine and Pharmacy, 3000041 Timisoara, Romania; man.dana@umft.ro (D.E.M.); dana.velimirovici@umft.ro (D.E.V.); olivia-maria.bodea@umft.ro (O.B.); daniel.duda-seiman@umft.ro (D.M.D.-S.); simona.dragan@umft.ro (S.-R.D.); 2 Research Centre of Timisoara Institute of Cardiovascular Diseases, “Victor Babes” University of Medicine and Pharmacy, 3000041 Timisoara, Romania; constantin.luca@umft.ro; 3 Center for Molecular Research in Nephrology and Vascular Disease, Faculty of Medicine, “Victor Babes” University of Medicine and Pharmacy, 300041 Timisoara, Romania; 4 University Clinic of Clinical Pharmacy, Communication in Pharmacy, Pharmaceutical Care, Department I, Faculty of Pharmacy, “Victor Babeş” University of Medicine and Pharmacy, 2 Eftimie Murgu Square, 300041 Timisoara, Romania; buda.valentina@umft.ro; 5 University Clinic of Cardiology II, Department VI—Cardiology, “Victor Babes” University of Medicine and Pharmacy, 3000041 Timisoara, Romania

**Keywords:** left atrial strain, speckle tracking echocardiography, diastolic dysfunction, diastolic function, HFpEF, left atrium, heart failure, biomarkers, diagnosis, echocardiography, exercise echocardiography, natriuretic peptides

## Abstract

Background: Heart failure with preserved ejection fraction (HFpEF) represents a significant portion of heart failure cases, but diagnosis is challenging due to its diverse presentation and the limitations of traditional echocardiographic parameters. Left atrial (LA) strain provides valuable insights into LA function and is increasingly used to evaluate cardiac function, including left ventricular (LV) diastolic function. LA strain, particularly reservoir strain, is considered a reliable indicator of LV diastolic function and can be used to grade diastolic function and estimate LV filling pressure. Unlike traditional LA measurements, LA strain offers detailed insights into LA function, conduit, and booster-pump phases, making it crucial for evaluating both structural and functional cardiac performance, especially in HFpEF. HFpEF diagnosis currently relies on a combination of echocardiographic parameters, clinical symptoms, and natriuretic peptide levels, encompassing various pathophysiological entities and complicating standardized management. Precise characterization of cardiac pathologies in HFpEF patients is essential. This review assesses global longitudinal strain (GLS) and left atrial strain (LAS) as echocardiographic biomarkers for diagnosing and characterizing HFpEF. Strain imaging, particularly speckle tracking echocardiography, offers a refined assessment of myocardial deformation, providing detailed insights into left heart function beyond traditional measures. Normal ranges for GLS and LAS are discussed, acknowledging demographic and technical influences. Clinical studies confirm the prognostic value of GLS and LAS in HFpEF, especially for predicting cardiovascular outcomes and distinguishing HFpEF from other dyspnea causes. However, variability in strain measurements and false-negative risks necessitate cautious clinical interpretation. The HFA-PEFF scoring system includes these biomarkers but does not fully cover the HFpEF pathology spectrum. Combining GLS and LAS shows promise in defining HFpEF phenogroups, potentially guiding individualized treatments. Global longitudinal strain (GLS) and left atrial strain (LAS) are central to non-invasive HFpEF diagnosis and stratification, with potential for more tailored therapies. Integration of these biomarkers into standard diagnostic practice requires an organized approach, and future guidelines should recommend their combined use for comprehensive HFpEF assessment.

## 1. Introduction

More than half of all heart failure (HF) cases are classified as heart failure with preserved ejection fraction (HFpEF) [1]. In patients presenting with HF symptoms, assessment of left ventricular (LV) function is typically conducted through transthoracic echocardiography (TTE), the primary imaging tool in clinical cardiology. Many HF patients with elevated or high-normal natriuretic peptide levels show a preserved or normal LV ejection fraction (LVEF) above 50% are currently categorized as HFpEF. However, HFpEF should be viewed not as a distinct diagnosis but rather as a syndrome. This phenotype, labeled “HFpEF”, can arise from multiple pathological entities, each with unique comorbidities and varied prognoses. Therefore, the true diagnostic objective of TTE in these patients extends beyond measuring LVEF; it is to identify underlying cardiac pathologies and achieve a precise diagnosis [2].

The term “diastolic HF” was originally defined as “an increased resistance to filling in one or both ventricles, causing symptoms of pulmonary congestion due to an abnormal upward shift in the diastolic pressure-volume relation”. This was later redefined as “HFpEF” [1]. Recent diagnostic algorithms classify patients with preserved LVEF and heart failure symptoms under the broad diagnosis of “HFpEF”, despite varying underlying pathologies, treatment options, and prognoses. These HFpEF algorithms rely on clinical symptoms, lab results, echocardiographic data, and/or invasive hemodynamic measurements, which often fall short of identifying the specific underlying diagnosis. When identifiable cardiac conditions such as valvular diseases, significant coronary artery disease, or pericardial constriction are found, they are classified as “HFpEF masqueraders” and should be excluded. Arrhythmias, often stemming from specific cardiac conditions, are also seen as HFpEF masqueraders, with the exception of atrial fibrillation (AF), which is frequently associated with HFpEF symptoms [2,3]. Ultimately, excluding HFpEF masqueraders may lead only to a diagnosis of HFpEF of unknown origin. Non-cardiac conditions—such as pulmonary diseases, anemia, diabetes, systemic infections, cancer, obesity, and frailty—add to the multifactorial complexity of HFpEF as a diagnosis [4,5]. In a study conducted by Shah A.M. et al. [6], it was documented that elevated right and left pressures, left ventricular hypertrophy, as well as right ventricular enlargement, all play a crucial role in outcome estimation. A recent study by Duchnowski et al. identified hs-TnT and NT-proBNP as independent predictors of postoperative cardiogenic shock requiring MCS in heart valve surgery patients. Given NT-proBNP’s role in HFpEF, these findings highlight the importance of hemodynamic monitoring and biomarker evaluation in HF risk stratification, linking myocardial stress and hemodynamic instability to both post-surgical outcomes and HFpEF [7].

The aim of this study was to analyze clinical, echocardiographic, and hemodynamic characteristics in HFpEF patients, focusing on parameters such as elevated right and left pressures, left ventricular hypertrophy, and right ventricular enlargement to identify key predictors of prognosis and refine diagnostic approaches.

## 2. Materials and Methods

From the beginning, the present review was designed to be a narrative review, with the main focus stipulated in the title of the present article (the clinical utility of left arterial strain in HFpEF patients). Two researchers conducted the literature research independently on two electronic databases (PubMed and Scopus) from June 2014 to September 2024. The search included different keyword combinations, such as “left arterial strain”/“speckle tracking echocardiography” AND “HFpEF”/“heart failure with preserved ejection fraction”/“diastolic function”/“diastolic dysfunction” AND “diagnosis”/“biomarkers”. Strain measurements were performed using vendor-independent software (Version 4.6, TomTec Imaging Systems GmbH, Unterschleißheim, Germany). A third researcher removed the duplicates using an Microsoft Excel (Microsoft Corporation, Redmond, WA, USA; Version: Microsoft 365) based on article title, author names, and year of publication. Moreover, the three researchers also screened the relevant review articles for additional references that may have been overlooked in the initial search.

### 2.1. Inclusion Criteria

The review included studies focusing on patients with chronic heart failure with preserved ejection fraction (HFpEF) and those with documented left atrial (LA) strain measurements using 2D speckle tracking echocardiography. Eligible study designs encompassed original research, meta-analyses, systematic reviews, and prospective and retrospective cohort studies. Only studies providing detailed insights into the relationship between left atrial strain (LAS), global longitudinal strain (GLS), and cardiac function were included. Outcomes of interest involved the evaluation of LA strain in the diagnosis, phenotyping, prognosis, or treatment response for HFpEF. Additionally, studies had to report echocardiographic parameters, left ventricular diastolic function, or predictive value for cardiovascular outcomes, such as major adverse cardiovascular events or atrial fibrillation. The review focused on studies utilizing 2D speckle tracking echocardiography as the primary method for assessing LA strain.

### 2.2. Exclusion Criteria

The review excluded studies that focused exclusively on heart failure with reduced ejection fraction (HFrEF) or other cardiac conditions without a specific emphasis on HFpEF. Pediatric or non-adult population studies were also excluded. Case reports, editorials, and narrative commentaries without primary data or comprehensive reviews were not included. Studies lacking quantitative analysis of LA strain or GLS were excluded. Articles that did not provide sufficient data on the role of LA strain in HFpEF or presented ambiguous or inconsistent strain values and thresholds were deemed ineligible. Studies using imaging techniques other than 2D speckle tracking echocardiography or those employing outdated or experimental imaging software without vendor-independent validation were excluded. Methodologically flawed studies, those with small sample sizes (fewer than 30 patients), or those providing low-quality evidence were omitted. Articles not published in English or those without accessible full texts were also excluded.

## 3. Discussion

Different stages and severities of heart failure (HF) present a wide range of echocardiographic findings, characterized by diverse functional analyses and varying strain thresholds. Among these, left atrial (LA) strain and left ventricular (LV) strain have emerged as critical early indicators of diastolic dysfunction and contractile impairment, often becoming evident before structural changes occur. LA strain reflects the atrium’s reservoir, conduit, and booster-pump functions, offering insights into atrial stiffness, elevated left ventricular filling pressures (LVFP), and early diastolic dysfunction [8,9]. Similarly, global longitudinal strain (GLS) of the LV is a sensitive marker for detecting subtle systolic dysfunction that may not be captured by traditional ejection fraction (EF) measurements. Together, these strain parameters play a crucial role in refining the phenotyping of HF, improving the identification of subclinical dysfunction, and guiding appropriate clinical management [8,9].

LA strain, in particular, has gained recognition as a valuable and increasingly reliable tool for assessing cardiac function, especially in the context of left ventricular diastolic dysfunction (LVDD) and heart failure with preserved ejection fraction (HFpEF) [8,9,10]. Its reliability stems from its capacity to provide detailed insights into left atrial function, which is intricately linked to LVFP. Studies have demonstrated that LA strain offers independent predictive value for elevated LV pressures [8,9] and serves as a sensitive marker for early diastolic dysfunction. Furthermore, it is non-invasive and reproducible, making it practical for routine clinical application. The use of 2D speckle tracking echocardiography (STE) to measure LA strain allows for real-time evaluation of myocardial deformation, offering a comprehensive view of atrial mechanics [10].

The strengths and weaknesses of LA strain are summarized in Table 1. Among its strengths, LA strain can detect subtle atrial dysfunction earlier than conventional parameters, such as LA volume, and provides significant prognostic value for heart failure progression, atrial fibrillation (AF), and valvular diseases. Additionally, it is a non-invasive and practical method that aids in risk stratification and tracking disease progression over time. However, LA strain is limited by technical variability across imaging platforms, dependence on image quality, and the lack of standardized cut-off values. Furthermore, irregular rhythms, such as atrial fibrillation, and significant mitral valve disease can impair strain measurement accuracy. Acute changes in loading conditions and variability in operator expertise can further complicate its application [10,11].

Despite its limitations, the strong correlation between LA strain and adverse cardiovascular outcomes, such as heart failure progression and atrial fibrillation recurrence, underscores its clinical utility. Research suggests that reduced LA strain is associated with worse prognoses, emphasizing its value as a prognostic marker [8,9,10].

LA strain is measured using 2D speckle tracking echocardiography, a non-invasive imaging technique that evaluates myocardial deformation by tracking the movement of acoustic markers within the echocardiographic image frame by frame. The process involves capturing high-quality echocardiographic images and analyzing them with software that calculates strain parameters. This method provides quantitative data on atrial mechanics, enabling clinicians to assess left atrial function and LV diastolic pressures with precision [10]. Although its reliability depends on operator expertise and technical consistency, LA strain remains a powerful tool for evaluating LVDD and HFpEF, particularly when integrated with other echocardiographic parameters [11]. With growing evidence supporting its prognostic value, LA strain is increasingly recognized as a cornerstone of advanced echocardiographic assessment, despite the need for further standardization and longitudinal research [8,9,10,11].

### 3.1. Characteristics of an Optimal Index for LV Diastolic Function

In patients with myocardial disease, there is often impaired left ventricular (LV) relaxation and increased LV chamber stiffness. An ideal index for assessing LV diastolic function should clarify whether it primarily reflects LV relaxation, LV chamber stiffness, or both. Although these two hemodynamic abnormalities frequently coexist, an optimal noninvasive index would primarily indicate one of these aspects while accurately representing the underlying abnormality it aims to measure, allowing for effective tracking of changes in either LV relaxation or stiffness over time [16].

To ensure reliability, this ideal index would minimize the influence of extraneous variables such as heart rate, LV systolic properties, and right ventricular–LV interactions. While it is challenging to develop a noninvasive measure that meets all these criteria, these attributes provide essential guidelines for evaluating potential indices of LV diastolic function. For example, some echocardiographic measures, like peak tricuspid regurgitation velocity, are not directly related to LV relaxation or stiffness. In contrast, indices such as myocardial velocities via tissue Doppler imaging (reflecting LV relaxation), myocardial diastolic strain rate (also reflecting LV relaxation), and shear wave propagation velocity (indicating myocardial stiffness) are directly impacted by LV diastolic function [14].

### 3.2. Measuring Left Atrial (LA) Strain

LA strain was initially measured using tissue Doppler imaging and is now primarily assessed with speckle tracking. The current guidelines recommend measuring LA strain in the apical four-chamber view, with the QRS complex as the reference point. Although LA strain can also be measured in the apical two-chamber view, which includes additional LA segments, there is no strong evidence suggesting this view provides added diagnostic or prognostic value [21].

**Figure 1:**
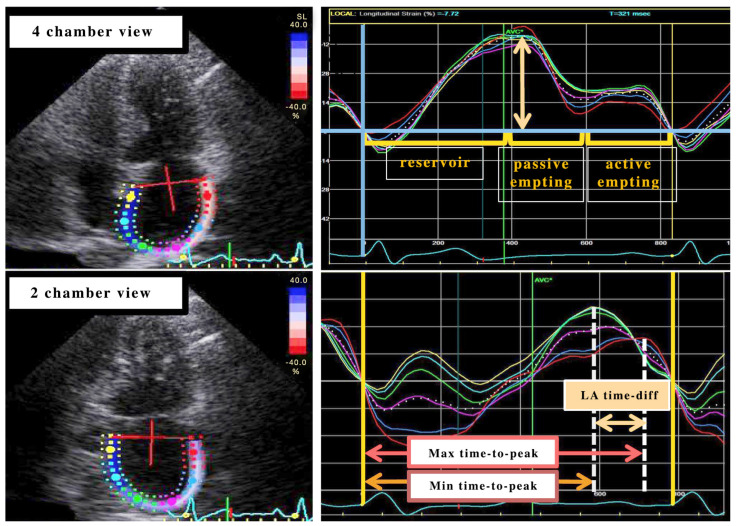
Echocardiographic analysis of left atrial (LA) strain: Phases of atrial function and dyssynchrony assessment.

**Figure 2:**
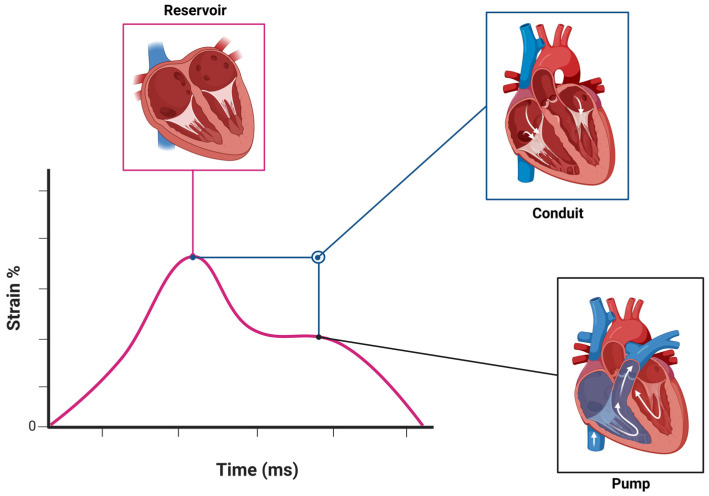
Phases of left atrial strain in HFpEF.

In practice, achieving clear imaging of the LA roof can be challenging, and significant variation in LA strain measurements may occur between the interatrial septum and the LA free wall. It remains unclear if the fewer segments captured in the apical four-chamber view yield comparable results to a comprehensive view with all segments, particularly in evaluating diastolic function [12] (Figure 1 and Figure 2).

Left atrial strain curve analysis: The right upper panel displays the left atrial (LA) strain curves from a normal subject. The LA strain curve consists of a positive peak during end-systole (reservoir phase), followed by two descending phases: one during early diastole (passive emptying) and the other during late diastole (active emptying). In the right lower panel, LA dyssynchrony is quantified as the maximum difference in time-to-peak regional LA strain, adjusted for the RR interval (referred to as LA time-diff). LA: left atrium; GLS: global longitudinal strain.

### 3.3. Normal Values of LA Strain

A recent important study that included 1765 normal subjects reported on the normal values of LA strain. There were 402 subjects older than 65 years of age, with 864 women. In this sample, 38.4% were White, 39.9% were Asian, and 9.7% were Black [13]. Strain measurements were performed by a vendor of independent software (Version 4.6, TomTec, Unterschleißheim, Germany). As noted, there is a wide range for normal LA strain values for all age groups and for both sexes. A study published by Singh et al. [22] is very important from our perspective because it includes both normal patients and patients with type 2 diastolic dysfunction, subjects from several countries, with a wide age range; here, central analysis was carried out in a core laboratory and using a software that is vendor independent. All these attributes are important, and as a result, when looking at normal values, we believe that the data from the report by Singh et al. [22] supersede findings based on single-center studies or meta-analysis that did not take into account the impact of the specific vendor software used to analyze LA strain. Importantly, other large and multicenter studies that reported on normal values of LA strain have shown similar findings with respect to lower limits of normal values for left atrial reservoir strain (LARS) [23].

### 3.4. Hemodynamic Influences on Left Atrial Reservoir Strain (LARS)

While there is strong interest in using LARS as an indicator of left ventricular diastolic function, data on its hemodynamic determinants remain limited. LARS, which occurs during systole, is influenced by various factors due to its association with left atrial (LA) filling. As LA strain reflects filling during systole, its relation to early systolic LA relaxation is crucial. Efficient LA relaxation, which occurs early in systole, reduces LA pressure, promoting forward pulmonary venous flow and increasing LA expansion [1].

**Figure 3:**
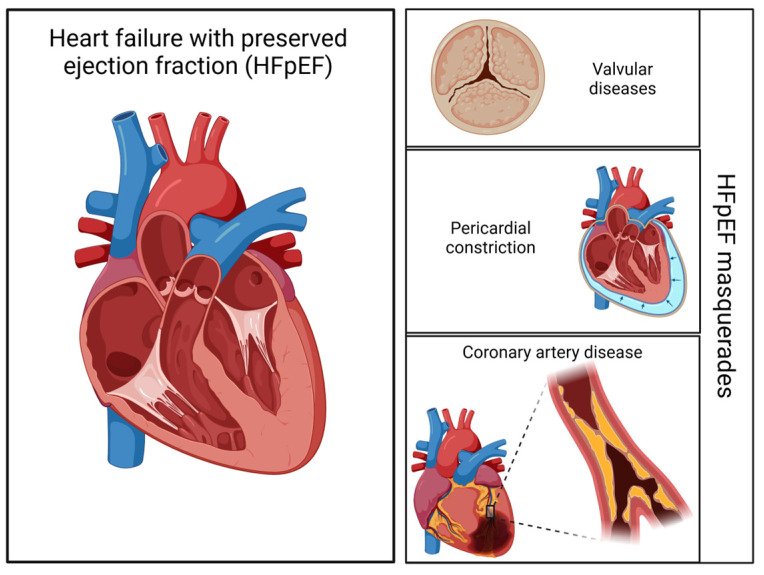
Alternative diagnoses mimicking HFpEF: key considerations for differential diagnosis.

Another key factor affecting LARS is the descent of the mitral annulus during systole, which is influenced by LV systolic function, especially the longitudinal function associated with sub endocardial fibers [2]. This movement can be quantified through LV global longitudinal strain (GLS). Additionally, LA chamber stiffness plays a critical role, as greater stiffness restricts LA expansion, raises LA V-wave pressure, and reduces the pressure gradient from the pulmonary veins to the LA, thus lowering LARS. Consequently, the relationship between LARS and LA pressures is indirect and modified by other hemodynamic factors [1] (Figure 3).

In studies involving patients undergoing cardiac catheterization, LARS showed a direct relationship with LA pump strain and LV GLS [2]. However, the association of LARS with mean pulmonary capillary wedge pressure and LV pre-A-wave pressure was significantly affected by LV systolic function. For patients with normal LV ejection fraction (LVEF), LARS proved less accurate in estimating LV filling pressure, and in patients with GLS ≥ 18%, no significant relation between LARS and LV filling pressure was observed. This may be due to an interaction where enhanced LA contractility supports faster LA relaxation, as evidenced by the relationship between LA reservoir and pump strain [18].

Further evidence of LA relaxation’s effects on LARS comes from a study by Pournazari et al. [17], which involved patients with primary mitral regurgitation undergoing transcatheter mitral valve repair. Here, LA relaxation was calculated as the pressure difference between peak A-wave and X-wave pressures relative to the time interval, showing that faster LA relaxation correlated with a more substantial pressure drop over a shorter time. This study confirmed a strong relationship between LA relaxation and LARS both before and after the procedure. Moreover, the study highlighted the influence of LA chamber stiffness on LARS, noting an inverse relationship between LA stiffness and LARS [24].

Through multiple regression analyses, LA relaxation, LV GLS, and LA chamber stiffness emerged as independent determinants of LARS before and after transcatheter mitral repair, although LARS variability post-repair was substantial [15,25].

### 3.5. Hemodynamic Influences on LA Conduit Strain and Pump Strain

While left atrial reservoir strain is often highlighted as a key indicator of LV diastolic function, less attention has been given to LA conduit and pump strain. Typically, impaired LV relaxation with normal LA pressure corresponds with reduced LA conduit strain and elevated LA pump strain. However, research in this area remains limited [15].

One study involving patients with hypertrophic cardiomyopathy found that LA conduit strain directly correlated with early diastolic mitral annulus velocity (e′). Conversely, LA conduit strain showed a significant inverse relationship with the myocardial extracellular volume fraction (measured by cardiac magnetic resonance) and with an index of LV end-diastolic pressure (LVEDP), namely, the time difference between atrial velocity in pulmonary vein flow and peak atrial mitral inflow (Ar-A duration) [26,27].

Thus, LA conduit strain appears to depend on both LV relaxation, as indicated by e′, and on LV interstitial fibrosis, as suggested by extracellular volume fraction, which impacts LV chamber stiffness (Table 2).

### 3.6. Determinants of LA Pump Strain

LA pump strain, occurring at end diastole, is influenced by LA systolic function and afterload, primarily reflecting LV late diastolic pressures. Early studies established a strong correlation between LA pump strain and LV filling pressures, a finding reaffirmed by recent research. Tayal et al. [28] demonstrated a direct relationship between LA pump strain and LA systolic function markers, including late diastolic mitral inflow peak velocity (A), mitral annulus late diastolic velocity (a′), and LA ejection force [29]. An inverse correlation exists between LA pump strain and Doppler-derived LV end-diastolic pressure (LVEDP), as represented by the Ar-A duration. Furthermore, LA pump strain correlates with LV late diastolic stiffness, measured via A-wave transit time. Increased LV stiffness shortens A-wave transit time, reducing LA pump strain due to elevated LV diastolic pressures [19].

### 3.7. Determinants of Left Atrial (LA) Pump Strain: HFpEF vs. HFrEF

LA pump strain reflects atrial contractile function and differs between heart failure with preserved ejection fraction (HFpEF) and reduced ejection fraction (HFrEF), influencing strain curve patterns and clinical interpretation.

HFpEF: LA enlargement and fibrosis from chronically elevated LV filling pressures increase atrial stiffness, reducing reservoir function. The booster pump phase compensates early, but progressive dysfunction decreases LA pump strain. A cut-off value <7–9% indicates significant dysfunction.

HFrEF: LA dilation results from LV systolic dysfunction and volume overload, leading to impaired contractility and remodeling. LA pump strain is markedly reduced, with a flatter strain curve reflecting severe dysfunction. A cut-off < 6–7% suggests advanced impairment (Table 3).

Clinically, LA pump strain serves as a marker of increased LA stiffness and subclinical dysfunction in HFpEF, while in HFrEF, it highlights advanced atrial remodeling and poor contractility. Disease-specific strain thresholds enhance heart failure phenotyping and management [27].

### 3.8. Correlation with LV Filling Pressure (PAWP)

Left atrial (LA) strain serves as a valuable non-invasive marker for assessing left ventricular (LV) filling pressures, commonly represented by pulmonary artery wedge pressure (PAWP). Abnormalities in LA strain, particularly during the reservoir phase, have consistently shown a strong correlation with elevated LV filling pressures, making it a useful tool for diagnosing and monitoring patients with HFpEF. A decrease in LV filling pressures is typically accompanied by a reduction in LA volumes, although complete normalization is rare. Importantly, there is a strong correlation between reduced LV filling pressures and improved LA function, as reflected by enhanced LA strain. This relationship is particularly significant, as elevated filling pressures are a hallmark of HFpEF and contribute to its clinical manifestations [30].

### 3.9. Association with Major Adverse Cardiovascular Events (MACE)

Left atrial (LA) strain has shown significant predictive value for major adverse cardiovascular events (MACE) in patients with HFpEF. Reduced LA strain is linked to worse cardiovascular outcomes, including all-cause mortality, cardiovascular mortality, and heart failure hospitalizations. The decline in LA function, as indicated by impaired strain, underscores the complex relationship between LA dysfunction and the progression of HFpEF. Consequently, LA strain serves as a valuable prognostic tool, helping clinicians in risk stratification and identifying patients at increased risk of cardiovascular events [31].

### 3.10. Risk of Atrial Fibrillation (AF)

Atrial fibrillation (AF) is highly prevalent among HFpEF patients and is associated with increased morbidity and mortality. Left atrial (LA) strain has emerged as a valuable predictor of AF development in this population. Reduced LA strain reflects impaired LA function, which is linked to atrial remodeling and electrical disturbances that facilitate the onset and maintenance of AF. As such, LA strain not only provides insight into the current status of HFpEF but also serves as a potential marker for identifying individuals at higher risk of developing AF [13].

In this context, left atrial (LA) strain plays a crucial role in the management of HFpEF by providing valuable insights into left ventricular (LV) filling pressures, predicting major adverse cardiovascular events, and identifying patients at risk for atrial fibrillation. As a key element of advanced echocardiographic assessment, LA strain has proven to be the most reliable imaging marker for distinguishing HFpEF from non-cardiac causes of dyspnea. Furthermore, abnormalities in LA strain are more strongly associated with adverse outcomes than those in LV function [20].

Abnormalities in left ventricular longitudinal strain have emerged as a valuable marker for identifying a distinct phenogroup within the heterogeneous HFpEF syndrome. This phenogroup, characterized by reduced global longitudinal strain (HFpEF-rLS), reflects the presence of contractile dysfunction, myocardial fibrosis, maladaptive hypertrophy, and other underlying myocardial disorders [19] (Table 4).

## 4. Future Directions

In patients with HFpEF-pLS and reduced LAS, there is a disproportionate impairment of left atrial (LA) function due to atrial cardiomyopathy, positioning the LA as a central mechanical hub in the disease’s pathophysiology. This perspective represents a relatively new concept in cardiology, as traditional understanding of diastology has primarily viewed LA dysfunction as a secondary consequence of advanced left ventricular (LV) diastolic dysfunction and chronically elevated LV end-diastolic pressure [32].

The development of left atrial (LA) strain imaging has highlighted its potential role in evaluating left ventricular (LV) diastolic function, providing additional insights beyond conventional echocardiographic indices. Strain analysis has demonstrated a strong association between reduced LV filling pressure and subsequent LA reverse remodeling with improved function, suggesting its future utility in clinical practice.

Numerous studies have explored the role of atrial strain imaging in heart failure, particularly in clarifying specific HFpEF subtypes where traditional diagnostic methods fall short. LA strain analysis may also prove valuable in assessing the effects of pharmacological interventions on atrial remodeling in heart failure patients. Additionally, the assessment of LA strain in coronary patients and hemodialysis patients with associated cardiac diseases could provide valuable insights into the broader applicability of this technique. In coronary patients, LA strain may help identify subclinical diastolic dysfunction, while in hemodialysis patients, it could offer unique perspectives on atrial remodeling under the influence of volume overload and chronic systemic inflammation. These patient populations represent critical areas for future research to extend the diagnostic and prognostic utility of LA strain imaging.

The implementation of LA strain imaging in the research and clinical management of atrial fibrillation (AF) is growing. Increasing evidence supports its utility in evaluating atrial remodeling, predicting thromboembolic risk, assessing the success of AF ablation, and identifying the likelihood of arrhythmia recurrence. However, larger studies are needed to validate these findings and determine whether strain analysis can reliably identify at-risk patients in routine clinical practice.

Additionally, efforts are underway to standardize atrial strain analysis across different imaging platforms and to develop new algorithms that are vendor independent.

By combining traditional HFpEF scores (HFA-PEFF, H2FPEF) with advanced imaging techniques (LA strain, GLS, cardiac MRI), stress testing, and biomarkers, clinicians can achieve a more detailed and accurate phenotyping of HFpEF. This multimodal approach improves diagnostic precision and aids in tailored treatment strategies.

## 5. Limitations

This study has several limitations that warrant consideration. As a narrative review, it does not follow the systematic approach of a meta-analysis, which could introduce selection bias and affect the generalizability of the findings. Additionally, there is considerable variability in the methodologies of the included studies, particularly in the techniques used to measure strain, such as vendor-specific software and imaging platforms. This inconsistency may lead to challenges in interpreting and comparing results across different studies.

A notable limitation is the lack of standardized cut-offs and reference values for left atrial strain (LAS) and global longitudinal strain (GLS), which complicates their clinical application. The patient populations across the reviewed studies are diverse, with varying comorbidities, demographics, and disease severities, further complicating the comparability of findings. The review also focuses primarily on echocardiographic assessments, largely excluding other imaging modalities like cardiac MRI, which might offer complementary or more detailed insights in certain cases.

There is also a risk of publication bias due to the restriction to English-language studies and the exclusion of gray literature, which may result in an overrepresentation of studies with positive findings. Moreover, the majority of studies included are cross-sectional or have short follow-up durations, limiting the ability to draw conclusions about the long-term prognostic value of LAS and GLS in heart failure with preserved ejection fraction (HFpEF).

The sensitivity of LAS and GLS measurements to loading conditions such as preload and afterload adds another layer of complexity, as these can vary significantly across clinical settings and patient populations. Operator dependency is also a concern, as the accuracy of strain imaging is influenced by the expertise of the operator and the quality of image acquisition. Furthermore, by excluding pediatric and younger adult populations, the study’s findings may not be applicable to these groups.

While some studies included in the review integrate biomarkers like NT-proBNP, the overall lack of extensive integration between strain imaging findings and biomarker data limits the potential for combined diagnostic or prognostic insights. Finally, the practical application of LAS and GLS in routine clinical practice has not been validated in large, multicenter prospective studies, leaving questions about their widespread feasibility and utility. Addressing these limitations in future research will be crucial to enhancing the clinical impact of strain imaging in HFpEF.

## 6. Conclusions

This review underscores the clinical utility of left atrial strain (LAS) as a precise and sensitive marker for evaluating left atrial function in patients with chronic heart failure with preserved ejection fraction (HFpEF). Evidence indicates that LAS correlates strongly with left ventricular filling pressures and diastolic dysfunction, offering a more nuanced assessment than conventional echocardiographic parameters. By providing early insights into atrial remodeling and dysfunction, LAS facilitates improved HFpEF phenotyping, which may ultimately guide targeted therapeutic strategies. Future research should concentrate on standardizing LAS measurement protocols and validating its prognostic value in large, multicenter cohorts, thereby enhancing its integration into routine HFpEF management.

## Figures and Tables

**Table 1:** Strengths and weaknesses of LA strain.

Strengths	Bibliography	Weaknesses	Bibliography
Early Detection of Dysfunction: LA strain can detect subtle left atrial dysfunction even before structural changes occur.	Aung et al., 2017 [12]; Nagueh and Khan, 2022 [13]	Technical Variability: Different imaging techniques, software platforms, and vendor-specific algorithms can produce inconsistent results, making comparisons challenging.	Khan et al., 2020 [14] Nagueh and Khan, 2022 [13];
Prognostic Value: Provides significant prognostic information for heart failure, atrial fibrillation (AF), and valvular diseases.	Kagami et al., 2024 [10]; Reddy et al., 2020 [11]; Shin et al., 2021 [15]	Image Quality: Poor echocardiographic windows, especially in obese patients or those with lung disease, can reduce the accuracy of LA strain measurements.	Aung et al., 2017 [12]; Nagueh and Khan, 2022 [13]
Sensitive Marker for LA Function: More sensitive than conventional parameters (e.g., LA size) in identifying reduced LA function.	Aung et al., 2017 [12]	Dependence on Heart Rhythm: Irregular rhythms, such as atrial fibrillation, can impair strain analysis and limit the reproducibility of results.	Reddy et al., 2020 [11]
Non-invasive: Offers a non-invasive way to assess atrial mechanics.	Ye et al., 2020 [16]	Mitral Valve Disease: The presence of significant mitral valve disease (e.g., stenosis, regurgitation, or annular calcification) can interfere with LA function, complicating strain interpretation.	Pournazari et al., 2022 [17]
Valuable for Risk Stratification: Helps identify high-risk patients in AF, heart failure, and post-surgery cases.	Maffeis et al., 2022 [18]	Load Dependence: LA strain is influenced by preload and afterload conditions, which may affect its reliability as a measure of intrinsic LA function.	Pournazari et al., 2022 [17]; Nagueh and Khan, 2022 [13]
Utility in Monitoring: Useful for tracking disease progression and treatment response over time.	Aung et al., 2017 [12]; Ye et al., 2020 [16]	Lack of Standardized Cut-Offs: There is no universally accepted threshold for differentiating normal and pathological LA strain values, leading to ambiguity in clinical practice.	Nagueh and Khan, 2022 [13]
Strong Correlation with Outcomes: LA strain correlates well with clinical outcomes like AF recurrence and cardiovascular events.	Jasic-Szpak et al., 2021 [19]; Shin et al., 2021 [15]	Limited Longitudinal Data: The prognostic value of LA strain over time and its role in disease progression remain areas of ongoing research.	Khan et al., 2020 [14]; Fauchier et al., 2023 [20]

**Table 2:** Hemodynamic reference values for LA conduit and pump strain in hypertrophic cardiomyopathy.

Reference	Patient Population	LA Conduit Strain (%)	LA Pump Strain (%)	Methodology	Key Findings
[15]	Hypertrophic cardiomyopathy patients	Reduced	Elevated	Speckle tracking echocardiography, echocardiographic indices	Impaired LV relaxation with normal LA pressure results in reduced LA conduit strain and elevated LA pump strain.
[26]	Hypertrophic cardiomyopathy patients	Correlated with e′	Not specified	Mitral annulus velocity (e′), cardiac magnetic resonance for fibrosis	Positive correlation of LA conduit strain with early diastolic mitral annulus velocity (e′); inverse relationship with myocardial extracellular volume fraction.
[27]	Hypertrophic cardiomyopathy patients	Inversely related	Not specified	Ar-A duration (time difference between atrial velocity in pulmonary vein flow and peak atrial mitral inflow)	LA conduit strain showed a significant inverse relationship with Ar-A duration, an index of LV end-diastolic pressure (LVEDP).

**Table 3:** Comparison of LA strain patterns in HFpEF vs. HFrEF [27].

Parameter	HFpEF	HFrEF
LA Reservoir Strain	Mild to moderately reduced	Severely reduced
LA Conduit Strain	Reduced (due to increased stiffness)	Reduced (due to LV dysfunction)
LA Pump Strain	Preserved initially, reduced later	Significantly reduced
Adaptation	LA compensates via active contraction	LA fails due to contractile dysfunction
Typical Cut-Off	LA pump strain < 7–9%	LA pump strain < 6–7%

**Table 4:** Articles published about left atrial strain.

Title	Authors	Year	Pts No	Study Type	Conclusion
Impaired Left Atrial Reserve Function in Heart Failure With Preserved Ejection Fraction [10]	Kagami, K.; Harada, T.; Yuasa, N.; Saito, Y.; Sorimachi, H.; Murakami, F.; Naito, A.; Tani, Y.; Kato, T.; Wada, N.; et al.	2024	240	Original research	Reduced left atrial (LA) reservoir function during exercise in heart failure with preserved ejection fraction (HFpEF) is linked to biventricular reserve limitations, exercise intolerance, and a higher risk of heart failure events.
Atrial Dysfunction in Patients With Heart Failure With Preserved Ejection Fraction and Atrial Fibrillation [11]	Reddy, Y.N.V.; Obokata, M.; Verbrugge, F.H.; Lin, G.; Borlaug, B.A.	2020	278	Original research	Left atrial (LA) compliance and mechanics progressively deteriorate as atrial fibrillation (AF) burden increases in HFpEF, elevating the risk of new-onset AF and the progression of existing AF.
Left Atrial Strain in Evaluation of Heart Failure with Preserved Ejection Fraction [16]	Ye, Z.; Miranda, W.R.; Yeung, D.F.; Kane, G.C.; Oh, J.K.	2020	450	Original research	Left atrial strain during the reservoir phase (LASreservoir) has the potential to identify patients with intermediate HFpEF scores who may exhibit elevated left ventricular filling pressures during exercise (LVFP-ex) only. Thus, it serves as a promising diagnostic alternative when exercise testing is not feasible.
Left atrial function in heart failure with preserved ejection fraction: a systematic review and meta-analysis [14]	Khan, M.S.; Memon, M.M.; Murad, M.H.; Vaduganathan, M.; Greene, S.J.; Hall, M.; Triposkiadis, F.; Lam, C.S.P.; Shah, A.M.; Butler, J.; et al.	2020	2725	Meta-analysis	Although impaired left atrial (LA) function shows potential diagnostic and prognostic value in HFpEF, its ability to significantly enhance diagnostic or prognostic accuracy has yet to be fully established.
Left atrial structure and function in heart failure with reduced (HFrEF) versus preserved ejection fraction (HFpEF): systematic review and meta-analysis [21]	Jin, X.; Nauta, J.F.; Hung, C.L.; Ouwerkerk, W.; Teng, T.K.; Voors, A.A.; Lam, C.S.; van Melle, J.P.	2022	18.734	Meta-analysis	While left atrial (LA) abnormalities have been proposed as a hallmark of HFpEF, our findings indicate that LA structure and function are more impaired in patients with HFrEF than in those with HFpEF. Therefore, the role of intrinsic LA myopathy as a key pathophysiological feature should be equally emphasized in both HFrEF and HFpEF populations.
Left atrial strain in heart failure with preserved ejection fraction [12]	Aung, S.M.; Güler, A.; Güler, Y.; Huraibat, A.; Karabay, C.Y.; Akdemir, I	2017	83	Prospective single-center cohort study	Left atrial (LA) function, as evaluated by 2D speckle-tracking echocardiography (2D-STE), is impaired in patients with HFpEF. A global left atrial strain during the reservoir phase (GLAs-res) value of <17.5% may serve as a useful diagnostic marker for HFpEF.
Left Atrial Strain for Assessment of Left Ventricular Diastolic Function: Focus on Populations With Normal LVEF [13]	Nagueh, S.F.; Khan, S.U.	2022			Left atrial (LA) strain is a valuable metric for assessing the mechanical properties of the LA, providing insight into its reservoir, conduit, and pump functions.
Heart Failure With Preserved Ejection Fraction: Do You Know Your Left Atrial Strain? [23]	Jellis, C.L.; Klein, A.L.	2016		Editorial	Beyond serving as an alternative prognostic marker in HFpEF, left atrial (LA) strain holds promise for tracking disease severity or treatment response over time. This could minimize the need for repeated comprehensive assessments of diastolic parameters during follow-up.
Left atrial strain predicts exercise capacity in heart failure independently of left ventricular ejection fraction [18]	Maffeis, C.; Rossi, A.; Cannata, L.; Zocco, C.; Belyavskiy, E.; Radhakrishnan, A.K.; Feuerstein, A.; Morris, D.A.; Pieske-Kraigher, E.; Pieske, B.; et al.	2022	171	Prospective single-center cohort study	In patients with chronic heart failure (CHF), impaired left atrial (LA) reservoir function is independently associated with significantly reduced peak oxygen consumption (pVO_2_). LA dysfunction serves as a marker of poor prognosis regardless of left ventricular ejection fraction (LVEF) classification within the CHF population.
Left atrial strain evaluation to assess left ventricle diastolic dysfunction and heart failure with preserved ejection fraction: a guide to clinical practice [24]	Reiber, J.H.	2023		Editorial	As highlighted in the authors’ summary, left atrial (LA) function plays a critical role in overall cardiac performance by modulating left ventricular (LV) filling through its three primary functions: reservoir, conduit, and booster pump. Due to the close interdependence between the LA and LV, LA size and/or function are often used as surrogate markers for LV diastolic function.
The novel left atrial strain parameters in diagnosing of heart failure with preserved ejection fraction [25]	Ma, C.S.; Liao, Y.P.; Fan, J.L.; Zhao, X.; Su, B.; Zhou, B.Y.	2022	389	Prospective single-center cohort study	The novel left atrial (LA) parameters could be valuable in estimating left ventricular filling pressure (LVFP) and, if integrated into the 2016 EACVI/ASE criteria, may enhance diagnostic efficiency. These parameters might also improve the ability to differentiate HFpEF patients from those with risk factors for HFpEF, increasing diagnostic accuracy.
Prognostic Value of Minimal Left Atrial Volume in Heart Failure With Preserved Ejection Fraction [15]	Shin, S.H.; Claggett, B.; Inciardi, R.M.; Santos, A.B.S.; Shah, S.J.; Zile, M.R.; Pfeffer, M.A.; Shah, A.M.; Solomon, S.D.	2021	347	Research article	In patients with heart failure with preserved ejection fraction (HFpEF), minimum left atrial volume index (LAVImin) was a stronger predictor of cardiovascular outcomes than indexed maximal LA volume. This suggests that LAVImin may be more physiologically relevant and better at identifying patients at high risk for cardiovascular events. Additionally, left atrial functional parameters offer prognostic information that is independent of LAVImin.
Prognostic Implications of Left Atrial Stiffness Index in Heart Failure Patients With Preserved Ejection Fraction [27]	Kim, D.; Seo, J.H.; Choi, K.H.; Lee, S.H.; Choi, J.O.; Jeon, E.S.; Yang, J.H.	2023	307	Retrospective single-center cohort	In patients with HFpEF, increased left atrial (LA) stiffness was associated with a higher risk of all-cause mortality and heart-failure-related hospitalizations. Moreover, its prognostic value exceeded that of traditional markers of left ventricular filling pressure.
Prediction of AF in Heart Failure With Preserved Ejection Fraction: Incremental Value of Left Atrial Strain [19]	Jasic-Szpak, E.; Marwick, T.H.; Donal, E.; Przewlocka-Kosmala, M.; Huynh, Q.; Gozdzik, A.; Woznicka, A.K.; Jankowska, E.A.; Ponikowski, P.; Kosmala, W.	2021	170	Original research	Peak atrial contraction strain (PACS) and peak atrial longitudinal strain (PALS) have predictive value for incident atrial fibrillation (AF) in HFpEF, beyond established clinical and echocardiographic predictors. Combining atrial remodeling markers, such as left atrial (LA) deformation and size, may provide a sensitive tool for screening AF risk in this population.
